# Growth rate controls the sensitivity of gene regulatory circuits

**DOI:** 10.1126/sciadv.adu9279

**Published:** 2025-04-25

**Authors:** Thomas Julou, Théo Gervais, Daan de Groot, Erik van Nimwegen

**Affiliations:** Biozentrum, University of Basel, and Swiss Institute of Bioinformatics, Basel, Switzerland.

## Abstract

Microbes adapt to their environments using gene regulatory switches that sense environmental signals and induce target genes in response. Mathematical modeling predicts that, because growth rate sets the intracellular dilution rate, the sensitivity of regulatory switches to chemical cues systematically decreases with growth rate. We experimentally validate that the concentration of inducer required to activate *E. coli*’s *lac* operon increases quadratically with growth rate when varying nutrients but is invariant when varying growth rate through translation inhibition. We further establish that this growth-coupled sensitivity (GCS) allows bacteria to implement concentration-dependent sugar preferences, in which a new carbon source is used only if its concentration is sufficient to improve upon the current growth rate. Using microfluidics in combination with time-lapse microscopy, we validate this prediction at the single-cell level using mixtures of glucose and lactose. Overall, GCS causes cells to automatically become more sensitive to environmental signals when their growth rate decreases.

## Teaser

Through the simple effect of dilution rate, regulatory circuits systematically change their sensitivity with growth rate.

## INTRODUCTION

From bacteria to humans, most biological organisms sense signals in their environment and use systems for processing this information to adapt their behavior. One challenge for such information processing systems is that optimal responses to environmental signals are often highly context dependent. For example, whether it is worthwhile to pursue a particular nutrient when detecting it in the environment will crucially depend on whether other, better nutrients are also available. While the central nervous systems of multicellular eukaryotes obviously enable complex context-dependent responses, it is now unclear to what extent bacteria are also capable of context-dependent responses to environmental stimuli, or how their relatively simple regulatory circuitry would implement such context dependence.

A number of studies over the past decade has shown that bacteria obey several so-called growth laws which determine how major aspects of cell physiology and proteome allocation vary with the growth rate during exponential growth (*1*–*4*). For example, it is well known that as growth rates increase, the absolute sizes of cells increase and, at high growth rates, DNA becomes polyploid (*5*, *6*). However, as the rates of biomolecular reactions inside cells are generally a function of molecule concentrations, we here focus on the scaling of protein concentrations with growth rate.

Although the global effects of these growth laws on protein concentrations are broadly understood for constitutively expressed genes and target genes in elementary circuits such as those of constitutively expressed activators (*7*), to what extent growth rate affects the responses of the regulatory circuitry of cells to signals from their environment has so far not been explored. Here, we use a combination of theoretical modeling and single-cell experiments with *Escherichia coli* to show that growth rate is a key contextual parameter for the response of regulatory circuits and controls their sensitivity to external signals.

The origin of this growth-coupled sensitivity (GCS) lies in the effects of dilution by growth. For any intracellular molecule that is stable relative to the doubling time of the cell, the rate at which its concentration decays is dominated by dilution and thus is equal to the growth rate. Consequently, whenever the rate of production of an intracellular molecule does not also increase at least proportional to growth rate, its steady-state concentration will decrease with growth rate. For example, for molecules that are imported into the cell by membrane-bound transporters at a constant rate and that are not actively degraded or metabolized, their steady-state concentrations will be inversely proportional to growth rate. Similarly, although transcription rates, mRNA decay rates, and translation all scale in a nontrivial manner with growth rate, the protein concentrations of constitutively expressed genes have been shown to decrease approximately inversely with growth rate (*2*, *7*). For other categories of genes, the situation can be more complex. For example, for genes involved in carbon catabolism, it has been shown that their protein concentration decreases linearly with growth rate when growth rate is modulated by carbon source quality but increases proportional to growth rate when it is modulated by translation inhibition (*3*).

The behavior of gene regulatory circuits is thus expected to be intrinsically coupled to changes in growth rate, since the concentrations of their protein players [e.g., transcription factors (TFs)] as well as of signaling molecules either activating or repressing them are affected by dilution due to growth. To explore such growth-coupled effects on regulatory circuitry, we focus on regulatory switches in which a positive feedback loop is coupled to a signal. These regulatory switches typically switch from an uninduced (“off”) to an induced (“on”) state when the intracellular concentration of the activating signal surpasses a critical concentration. Such regulatory switches are involved in many biological functions including the lysis-lysogeny switches used by phages (*8*), the regulatory circuitry involved in carbon source utilization (*9*–*11*), competence (*12*), sporulation (*12*), and virulence (*13*). In addition, a large fraction of the two-component signaling systems used by prokaryotes involves positive feedback loops and behaves as regulatory switches.

By exploring simple theoretical models, we show that GCS can be exploited by natural selection in a variety of ways. In the simplest scenarios, the result of increasing dilution rates is to decrease the sensitivity of regulatory switches so that faster growing cells are relatively insensitive, while slow growing or growth-arrested cells become hypersensitive to signals in their environment. However, by tuning the parameters of the regulatory circuitry, this default behavior can be modified or fine-tuned in a number of ways as we will explore below using the *lac* system in *E. coli*, which is arguably the archetypical example of a regulatory switch.

## RESULTS

We start by investigating how growth rate might affect the functioning of regulatory switches using mathematical models. We follow standard approaches for modeling simple regulatory circuits using deterministic coupled ordinary differential equations in which gene regulation is modeled by assuming that protein production rates are Hill functions of the concentrations of regulators, e.g., (*14*, *15*). However, in contrast to typical analyses of such models, we focus on the effects of dilution and explore how steady-state gene expression levels not only depend on extracellular concentrations of signaling molecules but also on growth rate.

**Fig. 1.**
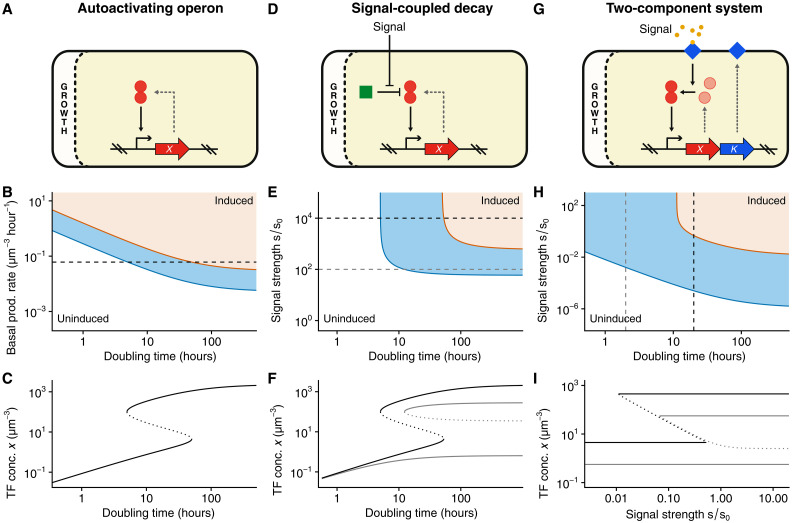
Theoretical analysis of gene regulatory switches exhibiting GCS due to growth rate–dependent dilution. (**A**) Minimal regulatory switch consisting of a TF (red circles) positively regulating its own expression, coupled to growth rate through dilution. (**B**) Phase diagram of the autoactivating TF expression rate depending on doubling time (white: uninduced; orange: induced; blue: bistable). (**C**) Induction curves showing TF concentration *x* as a function of doubling time for a basal expression corresponding to the dashed line in (B) (solid lines: stable steady states; dotted line: unstable steady state). The system is bistable for doubling times between 5 and 50 hours. (**D**) Regulatory switch in which an external signal represses the activity of a protease (green square) degrading an autoactivating TF. (**E**) Phase diagram for the regulatory switch with signal-coupled decay depending on doubling time and signal strength *s* relative to the signal strength *s*_0_ at which protease activity is at half maximum. (**F**) Induction curves showing TF concentration as a function of doubling time at high/intermediate signal strength, corresponding to the black/gray dashed lines in (E), respectively. At intermediate signal strength, the system remains bistable for arbitrarily large doubling times. (**G**) Two-component system regulatory switch. The autoactivating operon contains a TF gene *X* and membrane-bound kinase (blue diamonds) gene *K*. Kinase activity depends on an external signal and phosphorylation of the TF leads to its activation through dimerization. (**H**) Phase diagram of the two-component system depending on doubling time and signal strength. The minimal signal strength at which bistability starts decreases approximately quadratically with doubling time. (**I**) Induction curves showing TF expression as a function of signal strength at fast/slow growth, corresponding to the gray/black dashed lines in (H), respectively. At fast growth, the system remains bistable for arbitrarily high signal strengths. See the Supplementary Materials for detailed parameter settings.

For a minimal bistable regulatory system consisting of an operon containing a TF that activates its own expression, the parameter regime for which the system exhibits bistability has been shown to depend on growth rate (*7*). We here show that this simple positive feedback loop behaves as a regulatory switch as a function of growth rate, i.e., even without any coupling to an external signal (Fig. 1, A to C, and section S1.1). As growth rate is decreased, the operon goes from being stably switched off at high growth rates, to bistable at moderately slow growth, to being stably switched on at very slow growth. Moreover, the growth rates at which these transitions occur can be tuned by the basal expression level of the promoter (Fig. 1, B and C). Thus, the natural coupling to growth rate due to dilution turns a simple positive feedback loop into a regulatory switch that senses the cell’s growth rate. Although we are not aware of examples of regulatory switches that are solely controlled by growth rate, it is conceivable that phages could use such systems to switch on their lytic cycle as the growth rate of their host bacteria drops below a critical value.

In many known examples of regulatory switches, an operon activating its own expression is coupled to an external signal. For example, the regulatory circuits that implement competence and sporulation in *Bacillus subtilis* have at their core an autoactivating TF that is coupled to an external signal through repression of a protease that determines the TF’s decay rate (Fig. 1D) (*12*). The phase diagram of this type of circuit shows that it causes cells to only commit to sporulation/competence when both the external signal and the doubling time are over a critical value (Fig. 1, E and F, and section S1.2). Thus, although this regulatory circuit only interacts with a single signal, the coupling to growth rate makes the response of this system context dependent, effectively integrating two signals. Switching can occur either by varying the signal strength at a given growth rate or by varying the growth rate at a given signal strength. Moreover, the switching behavior as a function of one of these variables depends on the value of the other variable. For example, while at high signal strengths the system eventually stably switches on at sufficiently slow growth, at lower signal strength, the system remains bistable even at growth arrest (Fig. 1F), illustrating how such circuits can be tuned to function in one regime or the other. More generally, by tuning the parameters of the system, cells can implement stochastic responses in which only a subset of the population commits at intermediate growth rates or signal strengths. It is well established that competence is only induced in a subset of cells but not in the whole population, while sporulation occurs in the majority of cells (*16*).

The most common form of regulatory switches in bacteria involve two-component systems in which a TF positively regulates its own expression and is coupled to an external signal through phospho-relay by a membrane-bound kinase (Fig. 1G). In this simple example, we assume that the TF dimerizes upon being phosphorylated by the membrane-bound kinase, which, in turn, is activated by an external signal. In the parameter regime chosen for this example (see section S1.3 and fig. S1), the system switches from off to bistable and then to stably on as a function of signal strength at low growth rates (Fig. 1, H and I, black curves) but remains bistable for arbitrarily high signal strengths at high growth rate (Fig. 1, H and I, gray curves). In addition, the threshold level of the signal at which the system becomes bistable increases roughly quadratically with growth rate (Fig. 1H and section S1.3). That is, as doubling time increases by 10-fold, the threshold level of the signal decreases by 100-fold, showing that the sensitivity of the regulatory circuit strongly decreases with growth rate. This quadratic scaling roughly results from the fact that the concentrations of both the kinase and TF scale inversely with growth rate and holds as long as growth rate is large compared to the decay rate of these molecules.

While these three simple examples only scratch the surface of the possible ways in which growth rate can affect the functioning of regulatory circuits, they illustrate that theoretical modeling predicts that the natural coupling to growth rate through dilution can profoundly affect the sensitivity of gene regulatory switches.

**Fig. 2.**
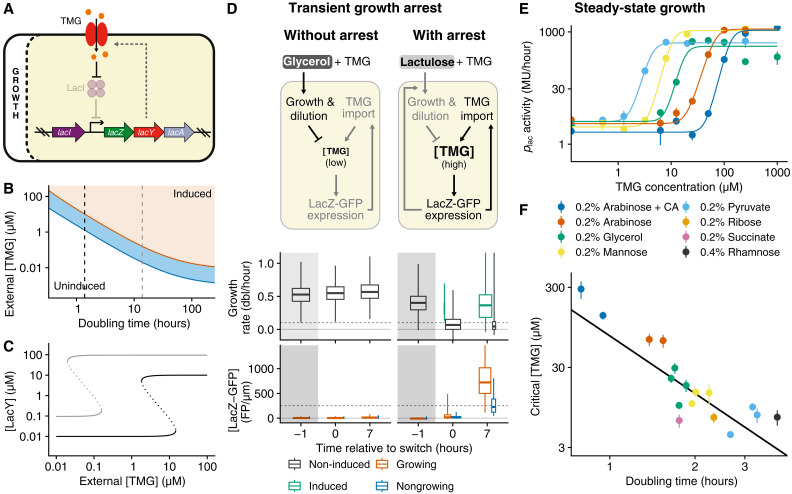
Growth-coupled sensitivity of the *lac* operon. (**A**) The *lac* operon’s positive feedback circuit. The intracellular concentration of the inducer, TMG, is set by LacY import and dilution by growth, leading to GCS. (**B**) Theoretical phase diagram of *lac* operon expression depending on doubling time and external inducer level (white: uninduced, blue: bistable, orange: induced; parameter values in the Supplementary Materials). The critical inducer level decreases quadratically with doubling time until doubling time becomes comparable to TMG and LacY’s half-life (assumed to be 48 hours). (**C**) Predicted LacY induction curves at a 1- to 2-hour doubling time (~growth on M9 lactose, black) and at 10-fold slower growth (gray). (**D**) Experimental validation that transient growth arrest increases TMG sensitivity. Cells growing in the DIMM microfluidic device without TMG are then exposed to 20 μM TMG and nutrients that either do not require *lac* operon expression (glycerol; left) or that do require the *lac* operon while not inducing it (lactulose; right). In the latter case, cells stop growing after the media switch because the *lac* operon is initially repressed. The boxplots show distributions of growth rates (top) and LacZ-GFP concentration (bottom) before, immediately after, and 7 hours after the switch (≥300 cells per time point). Cells were stratified by induction (top) or growth rate (bottom); see fig. S4. (**E**) Induction curves of the *lac* operon for cells growing in M9 media with different carbon sources and with locked-on CRP activity (using a Δ*cyaA* Δ*cpdA* mutant and 1 mM cAMP). Points and error bars show means and SE of three technical replicates for a representative biological replicate (solid lines: Hill function fits); see fig. S6 for all biological replicates. MU, Miller units. (**F**) The critical inducer concentration (estimated from fig. S6) decreases approximately quadratically with doubling time (exponent −2.4 ± 0.4), as predicted by our model (B).

To investigate experimentally whether regulatory switches in bacteria exhibit growth rate–dependent sensitivity, we here focus on the *lac* operon of *E. coli*. The *lac* operon consists of three genes which are involved in the metabolism of galactosides such as lactose. Its expression is repressed by the TF LacI and can be induced by lactose or artificial galactosides such as thio-methylgalactoside (TMG), which are not metabolized: When TMG is present, it inhibits DNA binding of the repressor LacI, leading to increased expression of the operon, including the transporter LacY, which increases transport of TMG into the cell, creating a positive feedback loop (Fig. 2A). Consequently, the *lac* operon can switch from an uninduced to an induced state when the TMG concentration exceeds a threshold value. We model the effects of dilution on the dynamics of this system by adapting a simple mathematical model of the *lac* operon (section S2 and fig. S2) (*9*). In the simplest scenario where the rate of *lac* protein production at full induction is independent of growth rate, the model predicts that the critical external level of TMG decreases quadratically with the doubling times of the cells as long as this time is short relative to the half-lives of the molecules (Fig. 2B). Intuitively, this quadratic dependence results from the fact that, in this regime, the intracellular TMG concentration is set by the ratio of import and dilution and that the import rate is itself also inversely proportional to growth rate, because LacY expression is also a balance between production and dilution.

This prediction reconciles observations in the literature that may have appeared contradictory. In particular, whereas Choi *et al.* (*17*) found critical levels of *lac* expression of several hundred molecules for cells growing in M9 media with glycerol and amino acids, our previous single-cell experiments show that, for growth-arrested cells, any nonzero *lac* expression is sufficient to induce the system when lactose is present (*11*). At parameter settings that match observations on the *lac* operon (section S2.3), the *lac* expression at the unstable steady state that separates the induced and uninduced states in the bistable region is predicted to be 0.1 to 0.2 μM (corresponding to a few hundred molecules) when cells are doubling every 1 to 2 hours, but at the same inducer level, cells are guaranteed to induce at low growth rates or growth arrest (Fig. 2C).

One prediction of our theory is that, because of GCS, inducer concentrations exist that are too low to cause induction in growing cells but that should cause induction when cells are growth arrested, even transiently. To test this prediction, we compare the responses of the *lac* operon to a given TMG concentration (20 μM TMG; see fig. S3A) in two different media. Using microfluidics in combination with time-lapse microscopy (*11*, *18*), we monitor growth and *lac* operon expression in single cells carrying a LacZ–GFP fusion at the native locus. Cells initially grow in minimal media with glycerol and are then exposed either to the same media supplemented with 20 μM TMG or to minimal media with lactulose and the same concentration of TMG (Fig. 2D). Notably, since lactulose requires LacZ to be metabolized, cells can only grow on lactulose if the *lac* operon is induced, but lactulose does not itself induce the *lac* operon (*19*). Under the first change in media, cells continue to grow on glycerol, and we observe that 20 μM TMG is not sufficient to cause induction of the *lac* operon in any of the cells during the entire experiment (Fig. 2D). A very different behavior is observed for the second change in media. First, since in the absence of inducer the *lac* operon is repressed during growth on glycerol, the change to lactulose causes almost all cells to immediately go into growth arrest, but several hours later, we observe that the large majority of cells have induced their *lac* operon and have recommenced growth (Fig. 2D and figs. S3 and S4). Thus, the transient growth arrest leads to the induction of the *lac* operon in most of the cells, demonstrating experimentally that cells become more sensitive to inducers of the *lac* operon when they are growth-arrested.

We next set out to test the prediction of the GCS theory that the critical inducer concentration increases quadratically with growth rate. However, our simple model of the *lac* operon assumes that the production at full induction is independent of growth rate, whereas the *lac* operon is known to also be regulated by the activity of the cAMP receptor protein (CRP), causing production at full induction to indirectly depend on growth rate. Below, we will explore the effects of this CRP regulation in detail, but to first test the simplest GCS model, we used a Δ*cyaA* Δ*cpdE* mutant strain. In this mutant strain, the CRP activity is kept constantly high because both the synthesis and degradation of cyclic adenosine 3′,5′-monophosphate (cAMP) are knocked out, and the 1 mM extracellular cAMP guarantees a constant intracellular level (*20*, *21*). It has previously been observed that, when cAMP-CRP activity is kept constant, the expression of the *lac* operon at full induction scales inversely proportional with growth rate (*22*).

To quantify how the critical external concentration of the inducer depends on growth rate in this mutant strain, we grow batch cultures in media with different carbon sources to modulate growth rate and, in each of the growth media, measure *lac* expression as a function of TMG concentration using the Miller assay (Fig. 2E and fig. S5). Fitting Hill functions to the observed induction curves (Fig. 2E and fig. S6), we estimate the critical external TMG concentration in each media. The critical concentration at which the *lac* operon induces decreases approximately quadratically with doubling time as predicted by the model (Fig. 2F and fig. S7), with an almost 100-fold change in critical concentration between the fastest and slowest growth conditions. This confirms that the sensitivity of the *lac* operon to its inducers depends strongly on the growth rate of the cells.

To confirm that the cAMP-CRP regulation does not markedly alter the sensitivity of the *lac* operon to growth rate, we perform analogous experiments with the wild type strain, using a strain carrying a LacZ-GFP fusion at the native locus and using fluorimetry to measure expression (see the Materials and Methods). We find that sensitivity decreases similarly with growth rate in the wild type as in the mutant of cAMP-CRP regulation (figs. S8 and S9). In addition, it is known that the activity of LacY transporters can be inhibited by other sugar transporters involving the phosphotransferase system (PTS) (*23*), and this “inducer exclusion” might also affect sensitivity of the *lac* system. However, using a mutant strain in which both cAMP-CRP activity is fixed and the *crr* gene is knocked out, we find that there is no systematic effect of inducer exclusion on either induction threshold or growth rate and that growth rate remains the key determinant of the induction threshold. Last, although it is of course impossible to fully rule out other growth rate–dependent mechanisms, the match between the experiments and our simple model supports that this growth rate dependence is mainly mediated through the effects of dilution (see section S2.4).

While the foregoing experiments establish that regulatory switches such as the *lac* operon exhibit GCS, it is unclear to what extent evolution has exploited this GCS to tune regulatory responses in an adaptive manner. For example, the current predominant view is that *E. coli* has a fixed hierarchy of preferences for carbon sources, so that when a mixture of such carbon sources is available, cells first exhaust the preferred carbon source before consuming the less preferred one (*10*, *24*). When grown in batch on a mixture of glucose and lactose, *E. coli* cells first consume glucose before starting to consume lactose. However, such a fixed hierarchy cannot always be optimal. For example, since it is well known that the growth rate that can be achieved on a given carbon source is a hyperbolic “Monod” function of its concentration (*25*), the growth rates that can be attained on different carbon sources crucially depend on their concentrations, so that it cannot be optimal to always prefer a given carbon source over another independent of concentration. It has thus long been clear that the optimal choice of carbon source should be concentration dependent, as has been noted by many others before us, e.g., (*26*, *27*).

Ideally, cells should opt for whatever carbon source maximizes growth rate in a concentration-dependent manner. That is, if cells are growing on carbon source *A* at rate λ_*A*_, and an alternative carbon source *B* appears at concentration *c*_*B*_, then cells should ideally only switch to consuming *B* if the growth rate λ_*B*_ that they can attain on *B* is larger than the current growth rate λ_*A*_. Since the growth rate λ_*B*_ increases with concentration *c*_*B*_, the minimal concentration *c*_*B*_(λ_*A*_) that is required to ensure λ_*B*_ ≥ λ_*A*_ is an increasing function of the current growth rate λ_*A*_ and given by the inverse of the hyperbolic Monod function for carbon source *B*.

As we have seen above, GCS does naturally cause the critical concentration for inducing the regulatory switch to increase with growth rate, and this raises the intriguing question whether GCS might be tuned so as to achieve such optimal carbon source switching. That is, to ensure that the critical induction concentration as a function of growth rate for a given carbon source matches exactly the inverse of the Monod function for that carbon source.

**Fig. 3.**
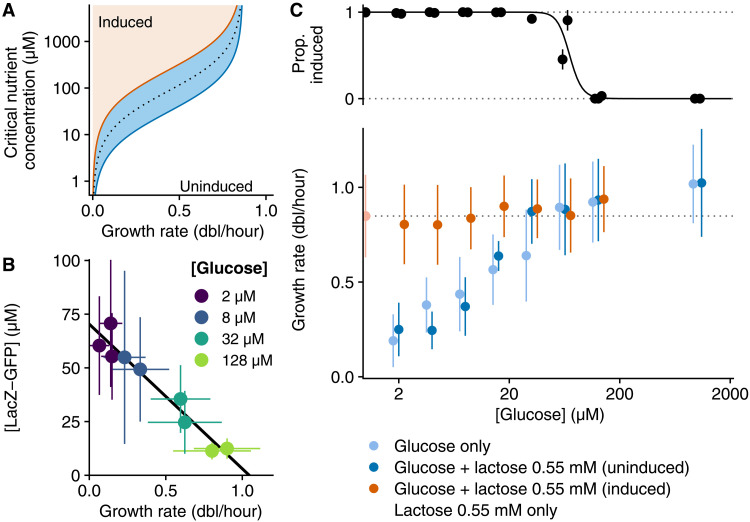
Growth-coupled sensitivity implements concentration-dependent sugar preferences. (**A**) Phase diagram of the *lac* operon regulatory switch when *lac* expression at full induction is regulated by CRP so as to decrease linearly with growth rate. The center of the bistable region (blue) perfectly tracks the inverted Monod function on lactose (dotted line). (**B**) Means and SDs of LacZ-GFP concentration and growth rate in single cells grown in media with different concentrations of glucose (in colors) and induced with 200 μM IPTG. The linear fit shows that fully induced *lac* expression decreases linearly with growth rate, as expected from CRP regulation. (**C**) Fraction of single cells with induced *lac* operon when growing on mixtures of saturating lactose (0.55 mM) and different glucose concentrations (2 μM to 1.11 mM) (top) and distributions of growth rates (mean ± SD) of the corresponding induced and uninduced subpopulations (bottom, orange and dark blue; note that even when the fraction of induced cells is close to 1 or 0, enough cells remain in both states to measure the distribution of growth rates). For comparison, the distributions of single-cell growth rates on media with only glucose at different concentrations (light blue) and on media with only lactose (light orange) are also shown. Bacteria were grown in a modified version of the DIMM microfluidic device where media is flown through the growth channel during the whole experiment (one to three independent replicates with more than 70 cells per condition; median ≈ 600 cells); no growth is observed in the absence of the carbon source (fig. S12).

As detailed in section S3 of the Supplementary Materials, we find that this is possible and is realized when the expression at full induction *y*_*h*_(λ) of the operon for the corresponding carbon source decreases linearly with growth rate λ as follows

$$y_{h}\left(\lambda \right)=y_{0}\left(1-\frac{\lambda }{\lambda_{*}}\right)$$ (1)

which should hold when growth rate is modulated by nutrient quality. In particular, when *y*_*h*_(λ) follows this form, it is possible for the critical external nutrient concentration to precisely track the inverse of the Monod curve provided that the parameters *y*_0_ and λ_*_ equal particular ratios of the kinetic parameters of the *lac* system (see eqs. S53 and S54 in section S3). The resulting optimal phase diagram is shown in Fig. 3A. Moreover, this behavior is also consistent with the relatively complex dynamics of LacZ converting lactose into the natural inducer allolactose and its hydrolysis of both lactose and allolactose (section S4 and fig. S10).

Recent work in the context of general bacterial growth laws has shown that cAMP and CRP regulate the *lac* operon in precisely the manner of Eq. 1, i.e., the expression at full induction decreases linearly with growth rate, reaching zero at a growth rate λ_*_ (*3*).

These theoretical results suggest that *E. coli* may be exploiting GCS to switch between carbon sources in a concentration-dependent manner such that the resulting growth rate is maximized. For example, in contrast to the current view that *E. coli* always prefers to consume glucose when grown on a mixture of glucose and lactose, the theory predicts that whenever the glucose concentration is sufficiently low, i.e., when the growth rate that can be attained consuming glucose is lower than the growth rate that can be attained consuming lactose, the *lac* operon will be induced.

Unfortunately, it is not possible to test such predictions using batch culture experiments, because substantial reductions in growth rate only occur when the glucose concentration becomes so low that cell densities in batch cultures are too small to quantify using standard techniques. We thus turn to microfluidics for testing these predictions. To ensure a homogeneous growth environment and avoiding nutrient gradients that would otherwise arise within dead-end channels at low nutrient concentrations (*28*), we modified our Dual Input Mother Machine (DIMM) microfluidic device such that growth media continuously flow through the channels in which the bacteria grow.

Using this device, we grow bacteria at different glucose concentrations ranging from the usual 0.2% (11.1 mM) down to 5500 times less, at a mere 2 μM. As the glucose concentration is varied, growth rates vary over a large range (from 1.06 down to 0.19 doublings/hour). Measuring fully induced *lac* operon expression at each glucose concentration by adding 200 μM isopropyl-β-d-thiogalactopyranoside (IPTG; which, in contrast to TMG, readily enters the cell without LacY), we find that average LacZ-GFP concentration decreases linearly with growth rate (Fig. 3B), confirming reports based on bulk measurements where the availability of glycolytic sugars was modulated by titrating their importers (*3*). Moreover, although there is much uncertainty about the kinetic parameters of the *lac* system, it is noteworthy that values predicted by our theory are consistent with our measured values of *y*_0_ ≈ 70 μM and that is 1.04 doublings/hour λ_*_ ≈ 0.72 h^−1^ (Fig. 3B; see section S3). Second, this constitutes, to our knowledge, the first experimental confirmation that growth rate increases as a Monod curve with glucose concentration also at the single-cell level (Fig. 3C, light blue symbols, and fig. S11).

Next we monitor growth and *lac* operon expression on mixtures of lactose and glucose, with glucose varying over the same range of concentrations and lactose at saturating concentration (0.55 mM, i.e., 0.02%) (Fig. 3C and figs. S12 and S13). When glucose concentrations are very low, almost all cells induce their *lac* operon and grow at rates similar to growth on media containing only lactose. However, although this is not visible in Fig. 3C, small fractions of cells with uninduced *lac* operon remain, and we find that their growth rates match the growth rates observed when growing on the same concentration of glucose only. As the concentration of glucose increases, the growth rates of the uninduced cells increases, and, around a glucose concentration of 50 μM, the distributions of growth rates of the induced cells and uninduced cells become virtually identical. It is exactly at this critical concentration that we also see the fraction of induced cells drop sharply, and at higher glucose concentrations, virtually, all cells are uninduced (Fig. 3C). That is, these single-cell experiments confirm our theory that, in contrast to the classical diauxie picture, there is no fixed sugar hierarchy, but *E. coli* induces its *lac* operon in a concentration-dependent manner so as to always grow on the carbon source that maximizes growth rate.

**Fig. 4.**
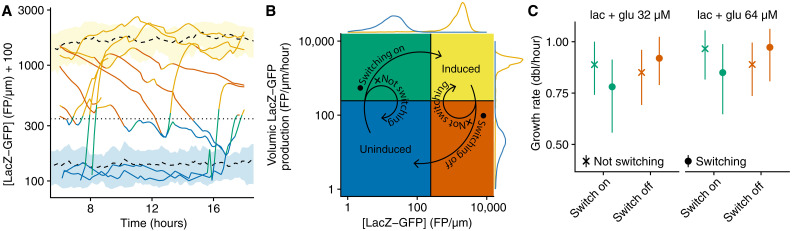
Growth-coupled sensitivity underlies the stochastic switching dynamics of the *lac* operon in single cells at intermediate lactose concentrations. (**A**) LacZ-GFP concentration traces of individual lineages dynamically switching their *lac* operon on/off in a mixture of lactose (0.55 mM) and glucose (64 μM). Colors correspond to the *lac* expression state as described in (B), and the horizontal dotted line shows the threshold between induced and uninduced cells (*e*^5.5^). Mean concentration ± SD of induced and uninduced cells is represented by the black dashed lines and yellow/blue ribbons, respectively. FP, fluorescent protein. For clarity, concentration values are offset by 100. (**B**) On and off switches of the *lac* operon are defined as trajectories in the LacZ-GFP concentration (*x* axis) versus volumic production (*y* axis) parameter space. Cells switching on go from the blue quadrant (low concentration; low production) to the yellow quadrant (high concentration; high production), while cells switching off are defined inversely (arrows with a dot). Cells whose production goes above or below the threshold but whose concentration remains below or above the threshold, respectively, are not considered switching (arrows with a cross). Thresholds are based on distributions measured for induced cells (yellow marginal distribution, lactose 0.55 mM) and uninduced cells (blue marginal distribution, lactose 0.55 mM + glucose 1.009 mM). (**C**) Comparison of the growth rate distributions of cells switching on/off versus cells not switching in the two glucose + lactose mixtures where growth rates of induced and uninduced cells are similar (lactose 0.55 mM + glucose 32 or 64 μM). Cell lineages observed in the green quadrant [low concentration; high production, see (B)] and orange quadrant [high concentration; low production, see (B)] are each sampled once and grouped according to the lineage fate (shown as symbol); points and error bars show median and interquartile range.

In the conditions where growth rates on glucose and lactose are near equal, i.e., at 32 and 64 μM glucose, our time-lapse microscopy measurements allow us to directly observe a notable number of events in which cells stochastically switch from uninduced to induced or vice versa (Fig. 4A). Such stochastic switches go through two phases. When a cell switches away from the uninduced state (blue area in Fig. 4B), it goes through a phase where the production rate of LacZ-GFP is already high, but the LacZ-GFP concentration is still low (green area in Fig. 4B). If the high production rate persists, then the cell will eventually move to the induced state with high LacZ-GFP concentration (yellow area in Fig. 4B), but sometimes, cells will return to the uninduced state (i.e., move from the green area in Fig. 4B back to the blue area). Similarly, for induced cells to switch off, they pass through a phase where the production rate is already low but LacZ-GFP concentration is still high (orange area in Fig. 4B).

Our time-lapse measurements also allow us to track fluctuations in the instantaneous growth rates of cells. If, as our GCS theory assumes, induction of the *lac* regulatory switch depends on growth rate through dilution, then this predicts that stochastic switches from uninduced to induced are more likely to be preceded by a transient dip in growth rate, whereas switches from induced to uninduced are more likely to be preceded by a transient increase in growth rate. To test this prediction, we identify all cells that moved into either the green or orange areas of Fig. 4B, stratify them by whether these cells eventually switch or return to their previous state, and compare the growth rates of these two groups of cells. As shown in Fig. 4C, we see that the predictions of our GCS theory are borne out in the single cell data: Cells that eventually switch on tend to have lower growth rates than those that return to the uninduced state, whereas cells that eventually switch off tend to have higher growth rates than those that return to the induced state (see also fig. S14). We cannot think of another interpretation of these results than that transient growth rate fluctuations contribute to stochastic switches of the *lac* regulatory switch.

These results on the *lac* system suggest that *E. coli* could more generally exploit GCS to implement a strategy by which induction of operons for alternative carbon sources is both growth rate and concentration dependent and tuned so that only the catabolic operon of the carbon source that is able to support the highest growth rate is induced.

Last, although above we have considered changes in growth rate due to changes in nutrient quality, growth rate can of course also be modulated by other environmental factors, e.g., by stresses that inhibit replication or translation. While, as discussed above, optimal concentration-dependent carbon source preferences require that critical inducer concentrations increase with growth rate, this should ideally only apply when growth rate is modulated by nutrient quality. In contrast, if growth rate is modulated by other environmental factors, critical concentrations should ideally remain unchanged. Such different behaviors as a function of how growth rate is modulated might appear difficult to reconcile with the GCS theory, because the dilution rate will be affected in the same way, independent of what environmental factors cause the change in growth rate.

One way to overcome this challenge is by modifying how the expression *y*_*h*_(λ) at full induction varies with growth rate depending on how growth rate is modulated. That is, while optimal concentration-dependent switching requires that *y*_*h*_(λ) decreases linearly according to Eq. 1 when growth rate is modulated by nutrient quality, we show in section S5 of the Supplementary Materials that if *y*_*h*_(λ) increases proportionally to λ when growth rate is modulated by other environmental factors, then the critical inducer concentration remains independent of growth rate. Studies on resource allocation in the context of bacterial growth laws have established that CRP regulation exhibits exactly this behavior. In particular, when growth rate is modulated by translation inhibition using sublethal doses of chloramphenicol (Cam), the expression of CRP targets increases in proportion to growth rate as shown in (*3*, *29*).

**Fig. 5.**
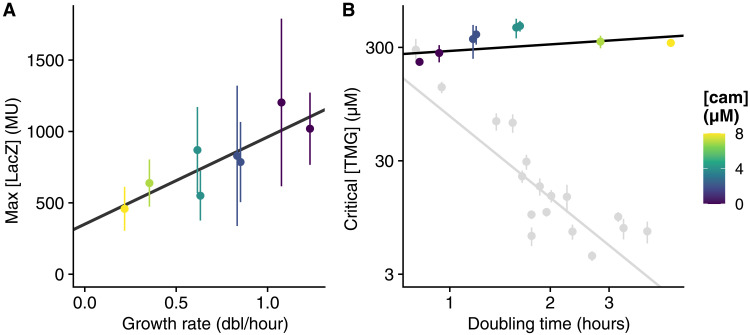
Critical inducer concentration is independent of growth rate when growth rate is modulated by translation inhibition. (**A**) Expression of the fully induced *lac* operon as a function of growth rate when cells are exposed to subinhibitory levels of Cam at different concentrations (in colors). (**B**) The critical level of TMG inducer is largely independent of growth rate when growth rate is modulated by Cam (colored points and black line for the linear fit). For comparison, critical TMG levels when growth rate is modulated by changing nutrients (same data as in Fig. 2F) is shown in light gray. In those experiments, 2 to 8 μM Cam were added to M9 + 0.2% arabinose + 0.1% casaminoacids; points and errors bars show the mean and SE of three technical replicates.

To test these final predictions of the GCS theory, we first use batch cultures and Miller assay to measure expression *y*_*h*_(λ) of the fully induced *lac* operon as we modulate growth rate using translation inhibition, i.e., starting from the highest growth rate condition (arabinose with casamino acids) and adding increasing levels of Cam. We confirm that *y*_*h*_(λ) increases approximately linearly with growth rate (Fig. 5A), although in contrast to (*3*, *29*), our measurements exhibit a nonzero offset at LacZ levels at zero growth rate. Since direct proportionality of protein levels to growth rate was reported not only for CRP targets in both (*3*, *29*) but also for constitutive genes in (*2*), we attribute this moderate quantitative discrepancy to imperfections of our Miller assay experiments.

We next use the same experimental approach as described above to measure *lac* promoter activity during balanced exponential growth and confirm the prediction that the critical external concentration of TMG is independent of the growth rate in this case (Fig. 5B and figs. S15 and S16). This confirms that CRP regulation not only optimally tunes the growth rate dependence of critical external inducer concentration under changes in nutrient quality but also ensures that this critical concentration remains unchanged when growth rate is modulated by translation inhibition.

## DISCUSSION

How does the functioning of gene regulatory circuitry depend on the growth rate of the cells? While studies of synthetic gene circuits have noted that their functioning is affected by growth rate (*30*, *31*), there has so far been no systematic study of how the responses of cells to environmental signals depends on their growth rate. Here, we explored in depth how growth rate affects the functioning of gene regulatory switches and how natural selection may have exploited this growth rate dependence.

The basic mechanism by which growth rate affects the functioning of gene regulatory circuitry is very simple and fundamental: A cell’s growth rate sets the rate of dilution of intracellular molecules, including those involved in gene regulation. Exploring this effect using simple mathematical models, we showed that growth rate generally affects the sensitivity of gene regulatory circuits. This GCS allows cells to respond to external signals in a context-dependent manner, and by tuning the parameters of the regulatory circuits, natural selection can exploit this GCS in a variety of ways.

For example, GCS allows cells to only respond to a particular signal when growth rate is below a critical value or to scale the critical level of an external signal with growth rate (Fig. 1). In particular, in the simplest default scenarios, gene regulatory switches decrease their sensitivity to their inducers with growth rate. For a basic model of the *lac* operon in *E. coli*, GCS theory predicts that critical inducer levels increase approximately quadratically with growth rate, and we verified these predictions experimentally using a mutant variant that is insensitive to cAMP-CRP regulation (Fig. 2). However, this basic behavior can be altered or fine-tuned by additional growth rate–dependent regulation. For example, it has been shown that while the maximal expression of carbon catabolism operons such as the *lac* operon decreases with growth rate when growth rate is modulated by nutrient quality, it increases proportionally to growth rate when growth rate is modulated through translation inhibition (*3*, *29*). Our GCS theory predicts that this causes critical inducer levels to be independent of growth rate when growth rate is modulated by translation inhibition, and we experimentally confirmed this prediction as well (Fig. 5B).

When multiple sugars are present in the environment at different concentrations, how do cells decide which sugar to consume first? Although the now predominant view is that there is a fixed hierarchy of sugar preferences, it is clear that optimizing growth rate requires concentration-dependent sugar preferences, because the growth rate that can be attained on a given sugar depends on its concentration. Arguably the most notable prediction of our theoretical analysis is that GCS provides a very natural way to realize optimal concentration-dependent sugar preferences.

In this general strategy, each alternative carbon source has its own regulatory switch consisting of a positive feedback loop coupled to its corresponding inducer and coupled to growth rate through dilution. Each feedback loop is tuned such that it will only induce if the concentration of the corresponding sugar is high enough such that, when switching to growth on this sugar, the growth rate will increase over the current growth rate. This ensure that when multiple carbon sources are present at different concentrations, only the positive feedback loop of the carbon source that (at its current concentration) provides the highest growth rate will be supercritical, and the positive feedback loops of all other carbon sources will be automatically switched off (fig. S17). In this way, only the catabolic genes for the sugar that maximizes growth rate at these concentrations will be switched on. Notably, this optimal strategy does not require any cross-regulation between the regulatory circuits of the different carbon sources.

We derived that to implement such an optimal strategy requires that cAMP-CRP signaling fine-tunes the full-induction expression of catabolic operons of different carbon sources to linearly decrease with growth rate. Notably, for the *lac* operon, precisely such a relationship has been observed previously (*3*), and we here confirmed that this relationship is consistent with the theoretically predicted optimal relationship (section S3 of the Supplementary Materials and Fig. 3B). In addition, using single-cell experiments with mixtures of glucose and lactose, we confirm that the critical concentration for *lac* operon induction is exactly where the single-cell distributions of growth rates on glucose and lactose match (Fig. 3C). Moreover, at glucose/lactose mixtures where the growth rates on glucose and lactose are very close, we find that stochastic on and off switches of the *lac* operon in single cells are accompanied by exactly the growth rate fluctuations predicted by our GCS theory.

It will be interesting to explore to what extent this regulatory strategy also applies to other sugar mixtures. In particular, it is well known that certain sugar mixtures are not used sequentially but in parallel (*24*), and we hypothesize that such situations might correspond to parameter regimes in which multiple regulatory switches are supercritical in parallel.

Although it is tempting to speculate that evolution may have tuned CRP regulation to specifically implement the optimal concentration-dependent sugar preferences that we observed here, it should be noted that the theoretical models that have been proposed to explain the so-called bacterial growth laws argue that the growth rate dependence of CRP regulation and even the Monod equation itself follow from the necessity to balance catabolic and anabolic intracellular fluxes (*2*, *3*). It is thus conceivable that the optimal concentration-dependent regulation of carbon source preference reported here is an emergent property of regulatory switches operating within the context of these growth laws. If this is the case, this would constitute a remarkable example of adaptive behavior emerging from more basic physical constraints.

Apart from the specific regulation of carbon source preferences that we investigated here, the fact that GCS causes regulatory circuits to typically become less sensitive with increasing growth rate is likely adaptive in general for bacteria. That is, GCS causes fast growing cells to stabilize their current state by effectively muting their response to fluctuations in external signals and causes slowly growing cells to become highly sensitive to external signals. This view is consistent with recent work from our lab that shows that gene expression noise in *E. coli* results to a large extent from the propagation of noise through the gene regulatory network and that noise levels systematically decrease with growth rate (*32*). This suggests a general strategy in which GCS causes slowly growing cells to more actively explore alternative gene expression states, and we have shown elsewhere that such behavior is highly adaptive for bet-hedging strategies (*33*).

The insights of our GCS theory may also have important applications in biotechnology, i.e., for the design of synthetic circuits. For example, the theory elucidates how different regulatory switches are globally coupled through dilution rate, and this insight might be exploited to design circuitry so as to induce the desired regulatory switches in a growth rate–dependent manner.

While we have here focused on the behavior of gene regulatory switches in bacteria, the coupling of gene regulatory circuits to growth rate through dilution is so general that it likely affects the operation of gene regulatory circuits across organisms, and GCS might also play a role in development and cell differentiation in multicellular eukaryotes. For example, since even a transient decrease in growth rate can cause regulatory switches to induce, it is conceivable that modulation of growth rate could be used in development to induce particular cell fate commitments. It seems that a mechanism of this type acts to control lymphoid and myeloid differentiation in mouse (*34*), and behavior suggestive of this mechanism has also been observed for the commitment to neurogenesis of neural progenitors (*35*, *36*). Exploring how GCS may have been exploited in the regulatory circuitry implementing the development and cell differentiation in multicellular eukaryotes is a fascinating area for future study.

## MATERIALS AND METHODS

### Bacterial strains and media

All strains used in this studies are derivatives of *E. coli* K12 MG1655. The strain used for microfluidic experiments is ASC662 (MG1655 *lacZ-GFP*mut2) (*19*), further characterized in (*11*). For induction experiments in a constant environment measured with the Miller assay, we used U486 (MG1655 Δ*cyaA* Δ*cpdA*) supplemented with 1 mM cAMP (growth rate modulated by different sugars) and MG1655 (growth rate modulated by subinhibitory levels of Cam). For induction experiments in a constant environment measured with LacZ-GFP fluorescence, we used U486 *lacZ-GFPmut2 and U486* Δ*crr lacZ-GFP*mut2 supplemented with 1 mM cAMP and MG1655 *lacZ-GFP*mut2 produced from the parent strain of U486 [Coli Genetic Stock Center (CGSC) #6300]. The integration of *lacZ-GFP*mut2 in the chromosome for these strains is described below.

All experiments were done using M9 minimal media (Sigma-Aldrich) supplemented with 2 mM MgSO_4_, 0.1 mM CaCl_2_, and sugars as indicated (typically 0.2% for glucose, lactose, or lactulose and 0.4% for glycerol). TMG and IPTG were diluted from frozen stocks in water (at 0.1 and 1 M, respectively), Cam from frozen stock in ethanol (0.1 M).

All experiments were carried out at 37°C. Note that the *melAB* operon is not expressed at this temperature (*37*) so that it cannot interfere with the regulation of the *lac* operon.

In experiments where the critical concentration of inducer as a function of growth rate was measured using fluorimetry, we used strains constructed from the MG1655 #6300 of CGSC. To integrate *lacZ-GFP*mut2 (taken from ASC662) into the chromosome of MG1655 (CGSC #6300) and of U486, we used the scarless in-frame insertion method developed by Cianfanelli *et al.* (*38*). To do so, we purified the pFOK plasmid from the diaminopimelic acid (DAP)–dependent strain JKe201, grown in LB supplemented with 100 μM DAP and kanamycin (50 μg/ml). We amplified the *lacZ-GFP*mut2-*lacY* region by polymerase chain reaction (PCR) using Q5 polymerase (New England Biolabs) with the primers oth21 (atgtaGCGGCCGCcaggaaacgccaataacatacag) and oth22 (atgtaCTCGAGgtaataagcgttggcaatttaaccg); flanking regions are composed of a spacer and a NotI/XhoI sequence. We conducted a restriction ligation between the PCR product and pFOK plasmid using NotI and XhoI restriction enzymes (New England Biolabs). We transformed the ligation product into a DH5α λpir strain with electroporation, amplified it, and purified it. We subsequently transformed JKe201 with the obtained ligation product and followed the protocol from (*38*), using MG1655 (CGSC #6300) and U486 as recipient strains. Note that the conjugation was done by concentrating the recipient and donor strain together and incubating them for 6 hours at 37°C on the edge of an agar plate.

Last, the strain U486 Δ*crr lacZ-GFP*mut2 was obtained by P1 transduction of the U486 *lacZ-GFP*mut2 strain obtained from the protocol above with a lysate of the Δ*crr* strain of the Keio collection (JW2410) (*39*). The integrations and deletions were checked in all strains using PCR and Sanger sequencing.

### Microscopy and image analysis

An inverted Nikon Ti-E microscope, equipped with a motorized xy stage and enclosed in a temperature incubator (TheCube, Life Imaging Systems), was used to perform all microfluidic experiments. The sample was fixed on the stage using metal clamps, and focus was maintained using hardware autofocus (Perfect Focus System, Nikon). Images were recorded using a CFI Plan Apochromat Lambda DM ×100 objective (numerical aperture 1.45; working distance 0.13 mm) and a CMOS camera (Hamamatsu Orca-Flash 4.0). The setup was controlled using μManager (*40*), and time-lapse movies were recorded with its multidimensional acquisition engine (customized using runnables). Phase contrast images were acquired using 100 ms exposure [CoolLED pE-100, full power]. Images of GFP fluorescence (excitation: 475/35 nm; emission: 525/50 nm; beamsplitter: 495 nm) were acquired using different exposure and excitation intensity (Lumencor SpectraX, Cyan LED) for different types of experiments as described later.

Image analysis was performed using the software MoMA as described in (*18*) and its documentation (*41*). Raw image datasets were transferred to a centralised storage and preprocessed in batch. Growth channels (GCs) were picked randomly for downstream curation using slightly different sampling schemes for the two series of mother machine experiments: For experiments on induction during transient growth arrest, at least 30 GCs were picked randomly for each experiment (after discarding channels with structural defects or no cells growing at the first switch of condition). For experiments on induction in sugar mixtures, the use of mother machine channels with shallow reservoirs on their sides (see the “Microfluidic experiments on the induction in sugar mixtures” section) led bacteria to eventually grow inside these reservoirs when nutrients concentration was low (typically less than 100 μM glucose), after what image analysis with MoMA was not possible anymore; since the delay until bacteria grow inside reservoirs is variable between experiments and between GCs, for each experiment and each condition, at least 10 GCs were picked randomly among GCs where cells do no grow early inside reservoirs and curated manually in MoMA.

MoMA’s default postprocessing was used to refine the measurements of total fluorescence. Fluorescence arbitrary units were converted to the number of GFP molecules using the procedure and conversion factors described previously (*18*).

### Microfluidic experiments on the induction during transient growth arrest

#### Experimental procedure

Experiments on the induction during transient growth arrest were done using the DIMM (*18*) controlled using a pressure controller (OB1 mk3, Elvesys), following a procedure described in detail in a previous study (*11*). Here, cells were exposed to 0.4% glycerol for 8 hours and subsequently to either 0.4% glycerol or 0.2% lactulose for 12 hours. To minimize phototoxicity, images were acquired only every 6 min (instead of every 3 min; with Lumencor SpectraX’s Cyan LED at 17% with ND4), and fluorescence excitation was decreased five times (400 ms instead of 2000 ms) when bacteria were exposed to lactulose and the induction threshold was changed accordingly in the corresponding analysis. For a list of the experiments carried out, see table S1.

#### Analysis of induction under transient growth arrest

For this experiment, it is critical to pick a TMG concentration slightly below the lower threshold of *lac* operon bistability, so that no induction happens in glycerol but that there is enough inducer to support growth on lactulose. We hence characterized induction by TMG for our strain (ASC662) growing exponentially in M9 + 0.4% glycerol. Cultures grown overnight from single colonies in M9 + 0.4% glycerol and diluted 100× in the same media supplemented with variable concentrations of TMG (50, 25, 12.5, and 6.25 μM). After 8 hours of growth, cultures were concentrated by centrifugation, plated on 1% agarose slabs (M9 without sugar), and imaged readily using the same settings as for the mother machine experiments. For each culture, the concentration of LacZ-GFP was measured in 400 bacteria as the average fluorescence per pixel (corrected for the 100 gray level offset of the camera in the dark) in a 5-pixel-wide circle close to the cell center. A simple graphical analysis revealed that 20 μM is the lower threshold of *lac* operon bistability in these conditions (fig. S3A).

As predicted, no induction happened in mother machine experiments with 20 μM TMG in M9 + 0.4% glycerol. To the contrary, when switched to M9 + 0.2% lactulose supplemented with 20 μM TMG, cells stop growing transiently and then induce their *lac* operon (Fig. 2D and fig. S3B), similar to the phenomenology under a switch to lactose (*11*). However, we note that the behavior on lactulose is more complex than on lactose. First, even in the absence of TMG, a fraction of the cells exhibited some growth and weak induction of the *lac* operon when growing on lactulose (fig. S4, A and B), possibly due to the very high activity of CRP and associated leaky *lac* operon expression in these conditions. In addition, because of the low concentration of extracellular TMG, even induced cells have relatively low steady-state levels of *lac* operon expression, i.e., two- to four-fold lower than on lactose (fig. S4A), and this made Lac protein expression limiting for growth as indicated by the strong correlation between instantaneous growth rate and LacZ-GFP level (fig. S4C). To verify that our analysis of the fraction of induced cells was done at steady state, we computed the distribution of induction lags using a procedure described previously (*11*) with a higher induction threshold (+1000 LacZ-GFP molecules instead of +200, to distinguish expression due to TMG from the weak expression observed in lactulose without TMG); this indicated that almost all cells which will ultimately switch do so within 5 hours (fig. S3B) and motivated our choice to estimate the fraction of induced cells between 7 and 8 hours. Overall, whereas no induction was observed in glycerol at all, the coupling to growth in the case of lactulose leads to the induction of the *lac* operon in the large majority (84%) of cells after 7 hours (Fig. 2D and fig. S4A).

### Measuring TMG sensitivity at steady-state growth using the Miller assay

The *lac* operon induction was measured in bulk for cultures in balanced exponential growth where the growth rate was modulated either by using different nutrients (0.2% ribose, 0.2% succinate, 0.2% rhamnose, 0.2% pyruvate, 0.2% mannose, 0.2% glycerol, 0.2% arabinose, and 0.2% arabinose + 0.1% casamino acids; referred to hereafter as “sugars experiments”) or by adding variable subinhibitory levels of Cam (2 to 8 μM) to M9 + 0.2% arabinose + 0.1% casamino acids (referred to hereafter as “Cam experiments”). The activity of the *lac* promoter at variable TMG concentrations was obtained using the Miller assay (*42*), which measures β-galactosidase enzymatic activity and was performed according to a protocol and analysis by Kuhlman *et al.* (*21*) with changes as follows.

Overnight cultures of bacterial strains were grown to saturation in 3 ml of M9 + 0.2% arabinose for sugars experiments or in 5 ml of M9 + 0.2% arabinose + 0.1% casaminoacids for Cam experiments. Cultures were diluted to 150 μl of into 96-well plates (Greiner) and grown for 16 hours in a humidity-controlled incubator (Cytomat 2, Thermo Fisher Scientific; shaking at 600 rpm with 1-mm radius). For each condition, M9 was supplemented with eight different concentrations of TMG to cover the induction range, and three combinations of dilution factor and delay before starting incubation were used so as to ensure having cells in mid-exponential phase after 16 hours. All cultures were grown at 37°C. Optical density at 600 nm (OD_600_) was measured every 20 min in a Synergy H1 plate spectrophotometer (Biotek) using an Orbitor RS (Thermo Fisher Scientific) for plate moving and delidding; the cell doubling rate (λ) was calculated for each sample as the slope of log_2_(OD_600_) versus time plot via linear regression analysis (figs. S7 and S15). When OD_600_ of all samples reached a value between 0.06 and 0.2 (corresponding to OD_600_ between 0.2 to 0.5 for a spectrophotometer with 1-cm light path), a 5× dilution of the sample in M9 without sugar nor Cam (0.1 ml in 0.4 ml) was transferred to a 2-ml 96-well polypropylene block containing 0.5 ml of Z-buffer (*42*), 20 μl of 0.1% SDS, and 40 μl of chloroform. All samples were thoroughly disrupted by repeated agitation with a multichannel pipettor. Two hundred microliters of each sample was transferred to a flat-bottom transparent 96-well plate (Greiner). Forty microliters of phosphate buffer containing *o*-nitrophenyl-β-d-galactopyranoside (4 mg/ml) was added to each well, and OD_420_ measurements were performed during 10 hours in a Synergy H1 plate spectrophotometer (Biotek), at 1-min intervals for the first 30 min and at increasing intervals (2 min during 90 min, 5 min during 8 hours) thereafter. Samples were maintained at 30°C throughout incubation, and the reader was preheated before loading the plate. All precultures and assays were performed in triplicate during each experiment, and one to four experiments were performed per condition (typically two).

Contrary to Kuhlman *et al.* (*21*), we did not observe an extended regime of linear dependence of OD_420_ on time but rather that the slope of this relationship first increases and then decreases between the start of the measurements and the maximum OD_420_ value (fig. S5A). To establish how to measure the β-galactosidase activity from such time series, we compared the activities estimated using different time windows (initial slope over 15 min, 30 min, 1 hour, 2 hours, 3 hours, or maximal slope) from culture samples containing variable concentrations of β-galactosidase. Practically, we mixed a culture induced with 500 μM TMG and an uninduced culture (so as to vary up to 10,000-fold the concentration of β-galactosidase per unit biomass) and diluted those samples to obtain three different cell concentrations (OD_600_ 0.8, 0.2, or 0.05). Since the apparent β-galactosidase activity is expected to increase linearly with the relative β-galactosidase concentration, we concluded that the activity is best measured using the initial slope (*s*) over 30 min, for concentrations as low as a few molecules of β-galactosidase (fig. S5B). Note that, at low LacZ concentrations, the OD_420_ sometimes started by decreasing during up to 1 hour (fig. S5A, right), in which case this initial decrease was discarded from the analysis. β-Galactosidase activity (*A*) was expressed in Miller units according to the formula *A* = (1000 *s*)/(0.5 OD_600_) (where OD_600_ was adjusted to be equivalent to using a 1-cm light path) (*42*), and the *lac* promoter activity (α) was estimated as α = *A* λ, where λ is the cell doubling rate defined above (in unit of 1/hour) and measured in each well. This measure of the promoter activity is motivated by the fact that the enzyme β-galactosidase is very stable so that in the balanced exponential growth, its “turnover” is governed by dilution due to cell growth (*21*).

For each condition, the TMG induction curve was fitted to the function

$$\alpha =b\;\frac{1+f{\left(c/k\right)}^{m}}{1+{\left(c/k\right)}^{m}}$$ (2)

where *c* is the TMG concentration, *b* the basal *lac* promoter activity, *f* is the induction fold change, *k* is the critical inducer concentration, and *m* is the Hill coefficient (Fig. 2E and figs. S6 and S16). While all other variables are almost independent of the growth rate, the critical inducer concentration *k* shows a clear positive correlation to growth rate when the growth rate is modulated by nutrients.

To fit the exponent for the dependence of the critical inducer concentration *k* as a function of doubling time *t* = 1/λ, we used all the estimated doubling times and critical concentration pairs (*t*_*i*_, *k*_*i*_) for all individual replicate measurements *i* and fitted a linear relationship *y*_*i*_ = *ax*_*i*_ + *b* to the logarithms $\left(x_{i}=\text{log}\left[t_{i}\right],y_{i}=\text{log}\left[k_{i}\right]\right)$. Note that, from the Hill function fits, we obtained an error bar σ_*i*_ for the relative error of the estimated critical concentration *k*_i_ of replicate *i*. We assume that the estimated *y*_*i*_ differ from those predicted by the linear relationship by both Gaussian measurement noise of SD σ_*i*_ and additional intrinsic deviation between model and experiment of SD σ. Given this model, the probability of the data given *a*, *b*, and σ becomes

$$P\left(D|a,b,\sigma \right)=\left[\prod_{i}\frac{1}{\sqrt{2\pi \left(\sigma^{2}+\sigma_{i}^{2}\right)}}\right]\;\text{exp}\left[-\sum_{i}\frac{{\left(y_{i}-{\mathrm{ax}}_{i}-b\right)}^{2}}{2\left(\sigma^{2}+\sigma_{i}^{2}\right)}\right]$$ (3)

We use a uniform prior over both *a* and *b*, a scale prior dσ/σ for σ, marginalize analytically over *b*, and maximize the resulting posterior over σ. From the resulting posterior of *a* (i.e., at optimal σ), we then lastly obtain the best slope *a* = −2.4 with an error bar (i.e., SD of the posterior distribution over *a*) of 0.4 (Fig. 2F).

In contrast to the strong dependence of the critical concentration on growth rate when growth rate is modulated by nutrients, no dependence on the growth rate was observed when it was modulated with subinhibitory levels of Cam (Fig. 5B).

### Measuring TMG sensitivity at steady-state growth using LacZ-GFP fluorescence

The *lac* operon induction was also measured in bulk using fluorimetry with a LacZ-GFP reporter at the native locus for cultures in balanced exponential growth where the growth rate was modulated by using different nutrients, i.e., M9 with 0.2% ribose, 0.2% pyruvate, 0.2% mannose, 0.2% glycerol, 0.2% arabinose, and 0.2% arabinose + 0.1% casamino acids. We took advantage of the fact that, in our hands, this assay is comparatively easier than the Miller assay, to measure induction in several different strains. In particular, we wanted to investigate the effects of native cAMP-CRP regulation and inducer exclusion on TMG sensitivity. We thus measured *lac* operon induction with TMG in U486 *lacZ-GFP* supplemented with 1 mM cAMP (to confirm consistency with the Miller assay), in the wild type MG1655 *lacZ-GFP* strain (to assess the effect of CRP regulation), and in U486 Δ*crr lacZ-GFP*. Notably, the *crr* deletion removes inducer exclusion, although it is also reported to affect CRP signaling (*23*, *43*).

Overnight cultures of bacterial strains were grown to saturation in 2 ml of M9 + 0.2% arabinose. Cultures were diluted (2000 to 10,000×) to 160 μl into black 96-well plates with clear bottom (Greiner). Plates were incubated at 37°C with continuous double orbital shaking (548 cpm frequency, “fast” speed) in a Synergy H1 plate spectrophotometer (Biotek), where OD_600_ and fluorescence (ex, 470 nm; em, 525 nm) were measured every 4 min. For each condition, M9 was supplemented (in duplicate) with eight different concentrations of TMG to cover the induction range, and several independent biological replicates were obtained for each condition.

We first background-corrected each growth curve by estimating background OD and fluorescence as the average value in a 20 time point window centered on the observation with minimal OD/fluorescence and subtracted these background values from all observations. Next, we identified a “growth segment” of each curve by finding the time point with maximum (background-corrected) OD and, going backward from this time point, find the segments of time points with background-corrected OD less then OD_max_/3, background-corrected OD larger than 0.01, and (background-corrected) fluorescence larger than zero. For quality control, we performed simple linear regressions of log OD and log fluorescence against time for the resulting growth segment and extracted their Pearson correlation coefficients. Among the 1600 growth curves, there was a small number (*21*) with anomalously little growth or poor Pearson correlations, and we removed these by hand. Growth segments with less than 21 time points were also removed. This left 1579 of the 1600 growth curves for further analysis.

Next, we extracted for the growth segment of each growth curve an average growth rate, an error bar on growth rate, an average log ratio *y* = log(fluorescence/OD) of background-corrected fluorescence and OD, and an error bar on this log ratio *y* as follows. We move a sliding window of length 21 across the growth segment and, for each window, perform a linear fit of log OD against time and log fluo against time. From these, we extract an estimated log ratio *y* with error bar and a growth rate λ with error bar. We then combine the estimated values of *y* and λ from each window into one final average *y* and λ (with error bars) for the growth segment.

Last, for each combination of a strain, media, and replicate, we extracted the estimated *y* values and their error bars for the series of growth segments with different TMG concentrations and then fit an induction curve exactly as described in the previous section using Eqs. 2 and 3. The resulting fits are shown in fig. S8. For each induction curve, we extracted the estimated critical TMG concentration and the average and SD of doubling time across the corresponding series of growth segments.

Figure S9 shows the observed relationship between doubling time and the critical TMG level for the U486 strain with constant cAMP-CRP (top left), the wild type MG1655 strain (top right), and the U486 Δ*crr* strain without inducer exclusion (bottom left). Symbols of the same color denote different replicates for the same growth media. It is notable that there is quite some variability across replicates and that, as indicated by the large error bars on the doubling times, growth rates are quite variable even within one replicate series. Nonetheless, the results for both the U486 strain and the wild type MG1655 strain are generally consistent with our results from the Miller assay experiments and are consistent with a quadratic decrease of critical TMG levels as a function of doubling time (black dotted lines).

We found that deletion of *crr* strongly affected the growth of the strains and their reproducicibility. Observed growth rates were often quite different from the growth rates of the other strains in the same media, e.g., with slower growth on arabinose with casamino acids than on arabinose. There was also very substantial variation in behavior across both technical and biological replicates, e.g., the large variation in growth rates in pyruvate. Although the critical TMG concentration still generally decreased with doubling time for the *crr* deletion, there is so much variation that no clear quantitative relationship can be inferred from these data.

Therefore, to quantify most directly the effect of inducer exclusion on the critical TMG concentration, we plotted the fold change in critical TMG concentration caused by the *crr* deletion against the fold change in growth rate caused by this deletion (fig. S9, bottom right). We find that in some media (i.e., arabinose, ribose, and glycerol), there is a little systematic effect on either induction threshold or growth rate. For mannose, the *crr* deletion leads to an increase in growth rate which is accompanied by an increase in critical TMG concentration, as predicted by our GCS theory. For pyruvate and arabinose with casamino acids, there is substantial variation in growth rate across replicates, and the change in critical TMG concentration correlates well with the change in growth rate, again supporting that dilution rate is a key determinant of the induction threshold. In summary, the results in fig. S9 show that inducer exclusion is not a key determinant of the induction threshold and instead support that growth rate is a key determinant of the induction threshold.

### Microfluidic experiments on the induction in sugar mixtures

#### Experimental procedure

##### Microfluidic device fabrication

The microfluidic device used in this series of experiments is similar to the DIMM device described earlier (*18*), with the main improvement that GCs are not closed on one end but rather designed to act like filters, letting media flow through but retaining cells. Given the small diameter of *E. coli* (typically between 0.6 and 1 μm in conditions considered here), it is very challenging to manufacture using ultraviolet soft lithography a constriction in the channel which is small enough to prevent cells from passing through and/or to block the flow through the GC. We solved this problem by modifying the initial design in two complementary ways: Each individual GC is surrounded by a large and shallow reservoir (width: 3 μm, depth: 0.25 μm, hence requiring a third microfabrication layer) as introduded by Norman *et al.* (*44*), and the back end of every reservoir is connected, via two small channels of the same depth as the GCs, to a back flow channel. This back flow channel is itself connected to an outlet to which a negative pressure is applied, thus generating a flow through the GCs running from the main flow channel to the back flow channel. Together, this allows media to flow through the GCs and their flanking reservoirs during the whole experiment, creating a homogeneous environment across the GCs, as indicated by the fact that the cell growth rate does not depend on the cell rank in the GC even at our lowest glucose concentrations of a few micromolars (fig. S12B). Notably, this is in contrast to experiments in classical mother machines where gradients in growth rate along the GC are observed, even at high concentrations of glucose (e.g., at 0.2%). This filter-like design of the GCs also greatly facilitates the cell loading step, which does not require concentrating the exponentially growing culture anymore, as the flow from the main channel through the GCs naturally drives cells into the GCs. Last, our device consists of eight independent series, allowing us to conduct up to eight different media switches with up to eight different strains in parallel. Because of space constraints, the mixing serpentines that existed in the original DIMM design had to be removed, so that only experiments with “hard” switches from one input media to another can be performed, i.e., precluding mixtures of the two input media.

Microfluidic masters were produced through soft lithography by Micro-resist GmbH; most of the data were produced using a single master with GCs and reservoirs of appropriate dimensions (GC, 0.8- to 1.0-μm width by 0.7-μm height; reservoirs, 3-μm width by 0.25-μm height). Device preparation was performed as described in the first series of microfluidic experiments, with two notable changes: Surface activation in the plasma cleaner was reduced to 10 to 30 s (typically 10 s) at 1300 to 1500 μm of Hg to prevent collapse of the reservoirs; the device was primed with bovine serum albumin (BSA; 10 mg/ml) from the back channel outlet and water from the overflow outlet. In these experiments, only one of the inlets at each “dial-a-wave” junction was punched, as we were not intending to perform condition switches.

##### Culture conditions and flow control

Bacteria were streaked onto LB agar plates from frozen glycerol stocks stored at −80°C. Overnight preculture was grown from single colonies in M9 minimal medium supplemented with 0.2% of glucose. The next day, cells were diluted 100-fold into fresh medium and harvested after 4 to 6 hours, at OD_600_ 0.05 to 0.15.

In the microfluidic devices, bacteria were grown in M9 minimal media with three different types of treatment: either glucose only at variable concentration, or a mixture of lactose (0.2%) and glucose (at different concentrations), or glucose (at different concentrations) supplemented with 200 μM IPTG to inhibit LacI repression.

For this, the experimental setup was prewarmed and initialized. The primed microfluidic chip was mounted, connected to the media supply, and flushed with running media for 30 min or more to rinse the BSA. Flow control was achieved using a pressure controller, applying 1500 mbar on the eight media inlets and −600 mbar on the back channel outlet. The resulting flow is approximately 4 to 5 μl/min through each flow channel. The exponentially growing bacteria culture was sucked (≈300 μl) using a syringe connected to the cell outlet, and all eight-cell–containing tubing pieces were connected to the pressure controller through a manifold and pressurized at 1800 mbar. This resulted in the cells being pushed into the flow channels of each series, and under the effect of the flow generated by the vacuum, the cells were aspirated in the GC very efficiently (15 to 30 min to load all eight series). It was necessary to disconnect the cell-containing tubing from the manifold once the series has been properly loaded to avoid clogging the flow channels.

After loading, the acquisition was quickly started. We typically acquired four positions per series, comprising ~40 GCs each. Phase contrast was acquired every 3 min with 100-ms exposure time, while GFP was acquired only every 9 min with low intensity (100-ms exposure time with SpectraX Cyan LED at 48%) to minimize phototoxicity.

#### Analysis of growth and induction

##### Population level growth and induction

In experiments with a mixture of lactose (0.55 mM, i.e., 0.02%) and glucose (at different concentrations), bacteria were precultured in M9 + 0.2% glucose where they grow fast (~1 doubling/hour), and the *lac* operon is repressed. Upon transfer into the mixture of glucose and lactose in the microfluidic device, they decrease their growth rate depending on the sugar concentrations and possibly induce their *lac* operon; note that, in most conditions, this induction is typically irreversible, i.e., the cell and its progeny will remain induced. To analyze how the fraction of induced cells and the growth rate depend on the concentration of glucose in the mixture, we needed (i) to set a criterium for calling a cell induced and (ii) to establish how long it takes for bacteria to adapt to the new condition so that we can measure variables at steady state. In the following, the single-cell growth rate is measured as the slope of the linear regression of log(length) versus time.

We first established that bacteria cannot grow on nutrient traces coming from the media or the setup (i.e., the microfluidic device, tubing, etc.), as indicated by the distribution of growth rate of cells exposed to M9 minimal media without carbon source (measured over windows of 1 hour; fig. S12A). To pick a criterium for induction, we examined time series of fluorescence in single cells, as well as distribution of LacZ-GFP levels at different glucose concentrations (fig. S12C) and observed that a threshold of 250 LacZ-GFP molecules clearly separates the two subpopulations at all glucose concentrations. In addition, we further checked that, using this threshold, virtually all cells were either induced or uninduced over their entire trace (0.8% of cells were induced more than 5% but less than 95% of their trace, and these were discarded from further analysis).

Looking at the fraction of induced cells as a function of time, we found that it stabilized within at most 5 hours and hence discarded the first 5 hours of each experiment. For concentrations of glucose close to the threshold of induction (32 and 64 μM), we noticed that it took longer to reach a stable fraction of induced cells and discarded the first 14 hours instead. Doing so, we discarded two replicates which terminated before 14 hours (32 μM glucose on 20210122 and 20210305; table S2). Moreover, examining the LacZ-GFP expression level revealed that one replicate was an outlier (probably due to an experimental mistake), and it was hence discarded from further analysis (16 μM glucose on 20210122; table S2).

To focus on cells for which growth rate can be reliably estimated, we filtered out cells which were monitored for less than 10 consecutive time points (30 min); note that this limit is below the shortest cell cycle that can be observed in our conditions, even at saturating glucose concentration. The same filtering criteria were applied to measure the distributions of growth rates when cells are growing in glucose only. The proportion of induced cells is reported for each replicate (Fig. 3C, top), while the distribution of growth rates is summarized per condition (Fig. 3C, bottom; mean SD); the corresponding histograms of growth rates are shown for each replicate in fig. S13.

We also set out to establish whether the linear dependency of CRP activity to growth rate reported in bulk (often called the C-line) (*3*) holds at the single-cell level when the growth rate is modulated by the sugar concentration. For this, bacteria were grown in glucose at different concentrations, each supplemented with 200 μM IPTG to inhibit LacI repression. Using the filtering criteria described above, we computed growth rate and the concentration (as the mean of the concentration measured over time) for each cell trace. Since the growth rate varies over a broad range, the fraction of GFP bleached due to repeated illumination is different between conditions, and the average diameter of the cell also varies. To estimate changes in diameter, we fitted an ellipse to each cell in GFP images for a few randomly selected frames and cells for each condition. Since the estimation of the diameter is noisy relative to its biological fluctuations, we did not intend to measure the diameter on each frame but rather estimated the average diameter in each condition as the average length of the ellipse’s small axis. For each observation, the cell volume was computed assuming a spherocylinder shape, using this average diameter and the instantaneous length of the cell. To estimate *c*, the concentration of GFP, from *c*_u_, the concentration of unbleached GFP, we assumed that the volumic production of GFP *q* and the growth rate λ are approximately constant over the cell cycle. At steady state, *c*_u_ = *q*/(λ + β), and the concentration of total GFP is *c* = *q*/λ, where β is the photobleaching rate. It follows that *c* = *c*_u_(1 + β/λ). Thus, for each cell, we estimated the average concentration of all LacZ-GFP molecules (bleached and unbleached) by correcting the average of the measured concentrations, with the photobleaching rate and the average growth rate. We report the joint distributions as the mean SD of growth rate and concentration for each replicate (Fig. 3B).

##### Single-cell level growth and induction

Glucose concentrations at which induced and uninduced cells coexist (32 and 64 μM) display interesting features: The growth rate achieved by induced and uninduced cells (which are expected to feed on lactose and glucose, respectively) is similar (Fig. 3C and fig. S13), and the fraction of induced cells takes longer to stabilize. Contrary to other conditions with more dissimilar growth rates, we observed that individual cells could switch the expression of the *lac* operon dynamically on and off during the experiments (Fig. 4A). We set out to establish whether those switches could be attributed to GCS at the single-cell level. In this context, the signature of GCS would be that cells switching on the expression of the *lac* operon typically grow slower than average before doing so and inversely for cells switching off.

In the previous section, we described the analysis of growth and induction at the population level, for which it was sufficient to estimate the growth rate and the induction status for whole cell cycle traces. To relate the growth rate to the induction dynamics at the single-cell level, it was necessary to get accurate estimates of the instantaneous growth rate, LacZ-GFP production rate, and concentration. To do this, our lab has developed a Bayesian procedure which uses Gaussian process priors for both the volumic production rate and the growth rate. The inference software RealTrace (https://github.com/nimwegenLab/RealTrace) first optimizes the Gaussian process prior by finding the combination of parameters (average, variance, and correlation times of both growth and volumic production rates, as well as the size of measurement noise in cell size and total fluorescence) that maximizes the likelihood of all the data of an experiment. With this maximum likelihood prior, the program calculates the posterior distributions over cell sizes, total fluorescence, instantaneous growth rate, and instantaneous volumic production rates for each cell at each time point.

We defined induced and uninduced states based on the distributions of concentration and volumic production observed in conditions where practically all cells are either induced (lactose 0.02%) or uninduced (lactose 0.02% + glucose 0.02%). On a logarithmic scales, both those variables are well separated by a threshold at 5.5. An on-switch of the *lac* operon expression was defined as going from a volumic production and a concentration below this threshold to both variables above the threshold and inversely for an off-switch, as illustrated in Fig. 4B. Therefore, a cell whose concentration would cross the production threshold not long enough for the concentration to increase above the threshold would not qualify as switching on and vice versa. In most cases, switches span over multiple cell cycles. We therefore considered switches based on lineages of three cells by appending, to every cell, the data from its parent cell and average from its daughter cells. The time of the switch was then defined as the time at which the volumic production rate of this lineage crossed the threshold (see time 0 on fig. S14A). Since the numbers of cells switching the expression of the *lac* operon dynamically are small compared to that of cells being stably induced or uninduced, we have analyzed 45 and 43 supplementary GCs in the 32 and 64 μM conditions, respectively. In total, we could analyze 332 cells switching on and 145 cells switching off in the 32 μM condition (2.8 and 1.2% of the cells) and 142 cells switching on and 81 cells switching off in the 64 μM condition (2.3 and 1.3% of the cells).

Switching lineages were aligned by the time of the switch to compute the average growth and volumic production rates as a function of time before and after the switch, using half hours windows around the switch. Comparing the growth rates of cells switching on/off to the average growth rate of induced and uninduced cells in those conditions showed that cells switching on the expression of the *lac* operon typically grew slower than average before doing so, while cells switching off the expression of the *lac* operon typically grew faster than average before doing so (fig. S14B). Similarly, comparing the growth rate distributions of cells switching and not switching in the two quadrants of the concentration versus volumic production rate space that correspond to transitions states between induced and uninduced, we could show that cells switching off the expression of the *lac* operon typically have a lower growth rate than the other cells that have high concentrations, while cells switching on the expression of the *lac* operon typically have a higher growth rate than the other cells that have a low concentration (see Fig. 4C and fig. S14B). This difference of growth rate is significant (*P* values are indicated in table S3 for a Welch two-sample *t* test). Together, those observations demonstrate that GCS affects induction dynamics at the level of individual cells and illustrate how this phenomenon shapes *E. coli*’s phenotypic heterogeneity in environments where no strategies is substantially better than the other.

## Supplementary Materials

### This PDF file includes:

Supplementary Text Figs. S1 to S17 Tables S1 to S3 References
